# Review on *Bifidobacterium bifidum* BGN4: Functionality and Nutraceutical Applications as a Probiotic Microorganism

**DOI:** 10.3390/ijms17091544

**Published:** 2016-09-14

**Authors:** Seockmo Ku, Myeong Soo Park, Geun Eog Ji, Hyun Ju You

**Affiliations:** 1 Department of Food and Nutrition, Research Institute of Human Ecology, Seoul National University, Seoul 151-742, Korea; sku@purdue.edu; 2 Laboratory of Renewable Resources Engineering, Department of Agricultural and Biological Engineering, Purdue University, West Lafayette, IN 47907-2022, USA; 3 Department of Hotel Culinary Arts, Yeonsung University, Anyang 430-749, Korea; mspark@yeonsung.ac.kr; 4 Research Center, BIFIDO Co., Ltd., Hongcheon 250-804, Korea; 5 Institute of Health and Environment, Graduate School of Public Health, Seoul National University, Seoul 151-742, Korea

**Keywords:** *Bifidobacterium*, functional foods, probiotics, nutraceuticals

## Abstract

*Bifidobacterium bifidum* BGN4 is a probiotic strain that has been used as a major ingredient to produce nutraceutical products and as a dairy starter since 2000. The various bio-functional effects and potential for industrial application of *B. bifidum* BGN4 has been characterized and proven by in vitro (i.e., phytochemical bio-catalysis, cell adhesion and anti-carcinogenic effects on cell lines, and immunomodulatory effects on immune cells), in vivo (i.e., suppressed allergic responses in mouse model and anti-inflammatory bowel disease), and clinical studies (eczema in infants and adults with irritable bowel syndrome). Recently, the investigation of the genome sequencing was finished and this data potentially clarifies the biochemical characteristics of *B. bifidum* BGN4 that possibly illustrate its nutraceutical functionality. However, further systematic research should be continued to gain insight for academic and industrial applications so that the use of *B. bifidum* BGN4 could be expanded to result in greater benefit. This review deals with multiple studies on *B. bifidum* BGN4 to offer a greater understanding as a probiotic microorganism available in functional food ingredients. In particular, this work considers the potential for commercial application, physiological characterization and exploitation of *B. bifidum* BGN4 as a whole.

## 1. Introduction

The term functional foods and/or nutraceuticals can be defined as certain foods reserving bioactive compounds that likely have beneficial effects in the body beyond basal nutritional ingredients (i.e., carbohydrate, protein and fat) [1,2]. According to USA Food and Drug Administration (FDA) [3], “terms such as functional foods or nutraceuticals are widely used in the marketplace and these are regulated by FDA under the authority of the Federal Food, Drug, and Cosmetic Act, even though they are not specifically defined by law”. Probiotics and probiotic foods are included within functional foods and have a growing market and large economic value [4]. The beneficial effects of probiotics on hosts beyond normal nutrition have attracted special interest from food industry and academia [5]. Functional properties of probiotic cells may offer various solutions to meet commercial demands for a variety of functional or conventional food products. Health benefits of probiotic cells coupled with consumers’ positive awareness regarding self-care, wellbeing and complementary medicine have reflected into multiple nutraceuticals as a promising ingredient in food industry [6,7]. Recently, multiple researchers have displayed interests to applications of food and nutraceutical processing to develop a novel concept of probiotic food or supplements [8,9].

Among the various probiotic bacteria, *Bifidobacterium*, is one of the most widely used and studied probiotic bacteria. According to Soto et al. [10], *Lactobacillus* and *Bifidobacterium* spp. accounted for 67.5% and 25.6% of microbial population, respectively, in breast milk (obtained from German and Austrian women, *n* = 160). Because the initial bacterial colonization is happening at an early stage of human life cycle, the primary colonization by breastfeeding or formula feeding has an important role to the individual health by affecting later host homeostasis during the development of the infant digestive and immune system [11]. Although, *Lactobacillus* is the major microbial flora in human milk, *Bifidobacterium* is the predominant cell species in fecal samples from breastfeed infants [12].

Naturally occurring microbiota in the intestinal tract of breast-fed infants, *Bifidobacterium* accounts for more than 80% of microorganisms within the intestine [13,14,15]. Among the various *Bifidobacterium* spp., *B. bifidum*, *B. breve*, *B. infantis* and *B. longum* are commonly detected bacteria from breastfed infants [12], whereas formula-fed infants have a complex ecosystem comprising mostly of coliform bacteria and *Bacteroides*, with significantly lower prevalence of *Bifidobacterium* spp. [16]. For marketing purposes, some food researchers in industry have tried to develop infant formula that stimulates *Bifidobacterium* spp. to become the dominant flora by constituting bifidogenic factors (e.g., non-digestible carbohydrates, and galactooligosaccharides) [17].

Recently reported studies showed that *Bifidobacterium bifidum*, (*B. bifidum*) is the second most prominent species that identified in breast-fed infants (the first was *B. breve* and the third was *B. longum*) [18]. As an early colonizer of the infant gut, *B. bifidum* is widely present among fecal microbiota, however, the concentration of overall *Bifidobacterium* spp. is decreased during the progression of age while *B. adolescentis* and *B. catenulatum* reach greater levels in adult guts [19]. Individual results from multiple studies had a little variation, however, it was clear that *B. bifidum* is considered a dominant species of gut population in healthy breast-fed infants. This distinctive ecological feature of *B. bifidum* spp. attracted microbiologists’ interests. Multiple experiments were carried out with clinical and pre-clinical studies, and proved significant health benefits (e.g., reducing bowel syndrome, diarrhea and pathogen infections) [20,21,22,23].

*B. bifidum* BGN4 (BGN4) *obtained from a* breast-fed *infant’s* fecal sample came to the forefront in 1996 by its distinctive enzymatic representation: β-glucosidase (E.C 3.2.1.21) negative [24]. This microorganism was first used to evaluate the expression of mutagenic activity by β-glucosidase-producing gut microbiota that produce deglycosyl hydrolases and catalyze carcinogenic glycosides (i.e., amygdalin, anthrone-6-*O*-rhamnoside, 8-hydroxyquinoline-β-d-glucoside, neocycasin A, quercetin-3-*O*-rutinoside, guercitrin, robinin, and cycasine).

BGN4 has been applied to multiple nutraceutical products and conventional foods in the global food markets (e.g., China, Germany, Jordan, Korea, Lithuania, New Zealand, Poland, Singapore, Thailand, Turkey, USA, and Vietnam) as a probiotic microorganism because of possible benefits to consumers [25]. Multiple researchers have proven outstanding bio-functional characteristics of BGN4 by in vitro, in vivo, and clinical experiments. Potential benefits of BGN4 include: (i) notable colon cell binding properties [26,27]; (ii) improved immune function [28,29,30,31,32,33,34]; (iii) anti-tumor effects [27,35,36]; (iv) aid in bioconversion of phytochemicals [37,38,39,40,41]; and (v) production of biogenic metabolites [42,43].

These represent the five main findings that have been discussed with regard to functional benefits from BGN4 with in-depth research. Optimizing cell culture conditions could increase not only BGN4 cell biomass recovery but also its bioactive metabolites. Recently, BGN4 chromosome sequencing was completed and will therefore be analyzed to further understand the correlation between genetics and physicochemical properties [44]. This review will highlight the importance of each of the five areas. In addition, this review will address prominent prototypes of BGN4 products, distinguished genome analysis and changed physicochemical attributes of BGN4 and the effect of altered culture conditions that were studied for the purpose of commercial manipulation.

## 2. Cell Adhesive Property

Foodborne illness (e.g., salmonellosis, listeriosis and shigellosis) occurs when food consumers eat a contaminated food with *Salmonella*, *Listeria*, and *Shigella* spp. [45]. Intestinal mucus and epithelial cells are predominantly susceptible to the attachment of pathogenic microorganisms resulting in active proliferation and colonization. This microbial adhesion is critical for the beginning of pathogen and epithelial cell interaction [46]. Consequently, avoiding bacterial adhesion onto the gastrointestinal mucosa is regarded as an efficient approach for decreasing the risk of foodborne disease [47,48]. Recently, Serafini et al. [49] observed inhibitory properties of *B. bifidum* PRL2010 against pathogenic bacteria (i.e., *Escherichia coli* and *Cronobacter sakazakii*) regarding enteric adaptation properties using epithelial intestinal cell monolayers (i.e., Caco-2 and HT-29).

Probiotic microorganisms are defined as “live microorganisms which when administered in adequate amounts confer a health benefit on the host” by WHO and FAO. Among the many probiotic strains, *Lactobacillus* and *Bifidobacterium* spp. are known as autochthonous microbiota in the human intestinal tract. These microorganisms have been used in various functional foods for centuries. The major functional effects that are provided by probiotics are: (i) production of anti-microbial peptides (i.e., bacteriocins) [50,51,52]; (ii) assimilation of dietary fibers [53]; (iii) regulation of fat storage [54,55]; (iv) modulation of mucosal immunity [56]; and (v) regulation of gut flora via competitive exclusion of pathogenic bacteria resulting in decreased pathogen colonization [57,58,59]. Among the five key functional effects of probiotics, attachment of probiotic bacteria onto the mucosal surface of the gastrointestinal tract is regarded as essential for the competitive exclusion of pathogens and must occur before effective regulation of immune activities, resulting in protective function against intestinal pathogens [60,61]. The cell adhesion stage of probiotics onto colon cells is essential for the successful microbial colonization inside of the host’s intestinal tract. This cell adhesion ability has been regarded as one of the critical screening standards for active probiotic strains [62], since adhesion is necessary to actively proliferate and provide a resistance to excretion from the intestinal tract as waste by peristalsis.

Mechanisms of bacterial adhesion onto epithelial cell can be divided into: (i) non-specific adhesion when regarding physicochemical factors of outer cell surfaces; or (ii) specific adhesion when considering the expression of specific molecules onto the microbial membranes that directly attach to the binding sites of epithelial cell mucosal surfaces [60,61,62]. This adhesion ability is a function of hydrophobic properties, level of ions, pH, and physical morphology [63]. These factors considerably affect microbial adhesion onto intestinal tissues of the host, demonstrating the complexity of preliminary microbial adhesion onto the mucosal surface.

According to Krasowska and Sigler [64], microbial hydrophobicity plays a key role in the initial interaction with the mucosal surface and epithelial cells of the intestinal lining due to the chemical composition of the bacterial surfaces. The physicochemical characteristics of the microbial outer membrane are generally estimated by analysis of cell surface hydrophobicity. It has been proven that microorganisms that express higher hydrophobicity more effectively attach onto the colon cells compared to hydrophilic microbial strains [61,62]. High cost and complexity of in vivo models encouraged attention into the use of an in vitro system for the initial selection and screening of potentially adherent probiotic microorganisms. Microorganisms that express high adhesive activity to inanimate surfaces (e.g., hydrocarbon surface) or non-polar solvents are considered hydrophobic, and cells that express lower adhesive activity are considered hydrophilic [62,64]. Pelletier et al. [65] reported that the existence of proteinaceous components on the microbial outer layer cause higher hydrophobicity, while hydrophilic properties are related to the existence of polysaccharides in the cell wall structure.

The hydrophobicity of strain BGN4 showed greater affinity towards xylene in similar studies (Table 1). Abdulla et al. [66] reported six different *Lactobacillus* strains with the hydrophobicity ranging from 29.5% to 77.4%. The three strains of *Lactobacillus* (i.e., *L. acidophilus*, *L. gasseri*, and *L. jensenii*) used by Boris et al. [67] showed about 80% surface hydrophobicity. *B. lactis* Bb12 and *L. acidophilus* LA5 showed surface hydrophobicity with values between 61% and 75% [68]. *B. pseudolongum* CIDCA 531 expressed 85% surface hydrophobicity [69]. Recently, Pan et al. [70] reported significant correlation between microbial adhesion property and cell hydrophobicity using 5 different *Bifidobacterium* strains (i.e., *B. longums* P-3, *B. animalis* H-9, *B. animalis* P-4, *B. asteroids* H-10 and *B. pseudocatenulatum* I-6) and Caco-2 with in vitro model. These results suggest that BGN4 may possess high cell adhesion properties and potent colonization abilities.

However, we should point out that the hydrophobic surface characteristics of probiotic bacteria do not consistently bind to epithelial colon cells [71,72]. The distinguished physicochemical surface of probiotics do not guarantee binding to epithelial colon cells. The adhesion property of microorganisms is significantly inconsistent and heterogeneous among cell strains [73]. Specifically, some probiotic strains show effective cell adhesion ability although they express significant hydrophilic properties on their cell surface [74]. This shows that other aspects that affect cell adhesion should also be considered. To overcome the limitation of cell hydrophobicity that often accompanies adhesion ability, microbial adhesion experiments using in vitro models with intestinal epithelial cells have been extensively *i*nvestigated [26,27,44,62,73]. The number of microorganisms attached to culture tissues directly shows the cell adhesion property. Among the various intestinal epithelial cell lines, the enterocyte-like Caco-2 cells obtained from a human colon have been routinely used to examine microbial adhesion mechanisms because of their distinctive physicochemical characteristics (i.e., active proliferation and differentiation under normal enrichment conditions, similar biological characteristics to normal enterocytes) [75].

**Figure 1:**
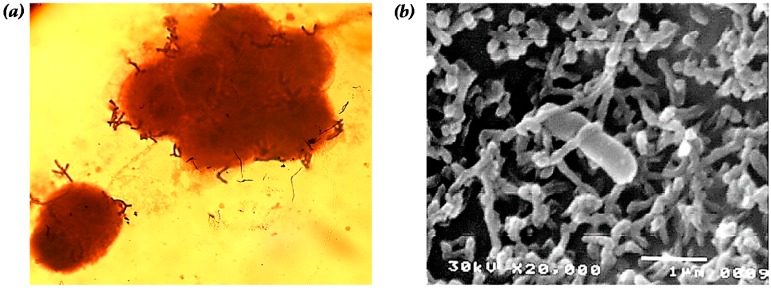
Adhesion of *B. bifidum* BGN4 onto the epithelial Caco-2 cell observed by: (**a**) optical (magnification of 1000×); and (**b**) scanning electron microscopy (magnification of 20,000×, interaction with microvilli of Caco-2 and *B. bifidum* BGN4). Microbial adherence in (**a**) was observed after simple staining with crystal violet. Panel (**b**) was adapted from Kim et al. [26].

BGN4 was compared to twenty different strains of *Bifidobacterium* spp. (i.e., *B. bifidum*, *B. animalis*, *B. adolescentis*, *B. infantis* and *B. longum*) separated from human fecal samples to evaluate cell adhesion properties [26]. According to Crociani et al. [76], cell adhesive properties of Bifidobacterium spp. are highly variable between strains of the identical genus. Kim et al. [26] clearly illustrated that binding between BGN4 whole cells and well-defined brush border microvilli on Caco-2 using scanning electron microscope (SEM). Among the various strains of *Bifidobacterium*, BGN4 showed the largest number of cells bound to the Caco-2 cells with highest cell surface hydrophobicity (93%) (Figure 1). Recently, 2.2 Mb of the BGN4 genome sequence was completely decrypted [44]. The comparative genomic analysis clearly elucidated the existence of a homolog (BBB_0596) of the *B. bifidum* MIMBb75 outer protein (BopA) that aids in the sticking of microorganisms onto a Caco-2 cell layer [44].

Despite this, very little research was done on specific adhesion related to the molecular mechanisms which possibly affects the strong adhesion properties, overall these findings evidently demonstrated the notable cell adhesive ability of BGN4 onto epithelial cell with its high hydrophobicity under in vitro conditions, and thus could represent better ability to colonize in the gastrointestinal tract with protracted transit. More detailed understanding of the: (i) adhesive mechanisms of BGN4 under the molecular level; (ii) fecal samples; and (iii) intestinal lining by biopsies could allow us to know the significance of adhesive ability of BGN4 and its applications to functional foods.

## 3. Immune-Modulatory Effects of *B. bifidum* BGN4

Multiple probiotic strains have shown significant bio-functional properties concerning boosted host immune functions. One of the important roles of probiotic bacteria is immune-modulatory activities for the prevention and regulation of multiple enteric diseases in the host [77]. According to Galdeano [78], orally consumed fluorescent-labeled probiotic cells were identified in the immune system (i.e., Payer´s patches and lamina propria mucosa) in the small intestine and lymphoid tissues (i.e., lymph nodules and colonic crypts). This report provides convincing evidence of a direct interplay between probiotic microorganisms and immune cells in the host’s intestinal lining.

Among the various immune cells, phagocytic cells (i.e., neutrophils, monocytes and macrophages) in the intestinal mucosa play an essential role in both stimulation of inflammatory responses against potential enteric pathogens and tolerance of normal colonic luminal nutrients and microbes as an innate immune system [79]. When macrophages are under an inflammatory stimuli, they generate cytokines, including Interlukin (IL)-1, IL-6, IL-8, IL-12, and tumor necrosis factor (TNF), which recruits other inflammatory cells. Phagocytic cells are attracted toward specific infection sites to engulf the opsonized targets using phagocytosis. They recognize pathogens using chemotaxis stimuli and/or straight physical connections [80,81,82]. Multiple reports have shown a significantly promoted phagocytic capacity of phagocytes by probiotic supplementation as an immunomodulator [83]. Therefore, the evaluation of the level of cytokines and macrophage activity using an in vitro assay is considered an indirect way of analyzing bio-functional effects of probiotic cells.

Lee et al. [28] reported the significant immunoregulatory capacities of whole-cell and cell-free extracts derived from BGN4. In this work, when macrophages were exposed to BGN4, active cell division, greater cytokine production and active phagocytic property were observed. Since then, various studies have focused on the interactions between outer cell wall and immune cells, however, little work that employs intercellular ingredients has been reported before. Therefore, they also extracted four different BGN4 cell fractions (i.e., whole-cell, cell free extracts, purified cell wall and supernatant) and treated cell lines to evaluate the level of cytokine produced by macrophages. As a result, each fraction showed different patterns of immune reactions. The whole cell fraction represented the strongest TNF-α expression. The cell-free extracts of BGN4 induced the highest IL-6 production.

This work was confirmed and further explained by Kim and Ji [29]. They made an attempt to determine the significance of type of BGN4 cell fractions with special focus on location within the host immune system. All BGN4 cell fractions (i.e., cell free extracts, whole cell fractions and cell wall fractions) significantly stimulated the production of IL-10 and IL-6. Cell free extracts of the BGN4 were able to induce greater morphological modification of macrophages with increased phagocytosis properties compared to macrophages treated with other BGN4 fractions (i.e., whole cell and cell wall fractions). The use of an in vitro assay clearly showed the immunomodulatory properties of BGN4 that activate differentiation of macrophages.

Another experiment was performed to examine the immune responses of intragastrically administrated BGN4 in a murine model of peanut allergy to provide further support function of BGN4 based on in vivo experiments [30]. They concluded that BGN4 treatment in an animal model showed anti-allergic and immunomodulatory effects by decreased levels of peanut-specific IgE and IL-4 and increased levels of IL-12 and the ratio of Interferon (IFN)-γ/IL-4. Kim et al. [31] also reported clinical properties of BGN4 to inflammatory bowel disease using a mouse model. The BGN4-fed group showed minimal signs of thickened wall and inflammatory cell infiltration, in a clinical sense, such as: (i) thickened wall; (ii) crypt elongation; (iii) reduction of goblet cells; and (iv) maintaining the level of cluster of differentiation (CD) 69, IFN-γ, TNF-α and MCP-1 in the mouse intestine than its counter group. The in vivo approaches employed in this work clearly suggest further functional characterization of BGN4 on the control of the aberrant intestinal immunity.

However, an additional question is: does BGN4 show potent immune stimulating effects within clinical experiments? This answer is critical to prove practical benefits of BGN4. Despite various in vitro, in vivo data, the precise mechanism of action of BGN4 was not fully demonstrated and could be multifactorial in clinical research.

According to Hong et al. [32], a probiotics mixture containing 2 × 10^10^ of lyophilized cells (i.e., BGN4, *B. lactis* AD011, *L. acidophilus* AD031and *L. casei* IBS041) was effective to relieve irritable bowel syndrome. They randomly divided two groups (*n* = 36 and 34; age: 19–75 years; sex: male and female; symptoms: presence of previous gastrointestinal disease) as probiotics and placebo groups, respectively. Their work clearly demonstrated that probiotics treatment was statistically significant in the reduction of abdominal pain and defecation discomfort after eight weeks of probiotics treatment compared to placebo groups (*n* = 70, −31.9 vs. −17.7, *p* = 0.045), and concluded “composite probiotics containing BGN4, *L. acidophilus* AD031, and other species are safe and effective, especially in patients who excrete normal or loose stools”.

Kim et al. [33] used different strategies to characterize functional effect of BGN4 for their role in eczema. Through the randomized, double-blind and placebo-controlled experimental design (*n* = 112, screened and randomized pregnant women having family history of allergic diseases), they evaluated the preventive function of BGN4 against progress of eczema. They concluded that the prevalence of eczema can be statistically significantly decreased by BGN4 treatment compared to its counter placebo group (completed sample number: *n* = 68, *p* = 0.048, BGN4 group: 18.2% vs. placebo: 40.0%). Interactions among gut microbiota, intestinal epithelial cells and mucosal dendritic cells in the lamina propria, and their impact in innate immunity has been the focus of multiple researchers in recent decades [84]. Specifically, Kim et al. [34] discussed function of BGN4 treatment into the dendritic cells. With comparison of cell culture conditions (i.e., single culture of dendritic cells or co-culture of dendritic cells and mouse epithelial cell monolayers), multiple conditions for exerting immune-modulatory reactions were evaluated. The authors concluded that BGN4 significantly upregulated the expression of I-Ad and cluster differentiation (i.e., CD86 and CD40) (*p* < 0.05) with increased secretion levels of pro-inflammatory cytokines (i.e., IL-6 and TNF-α). These results indicate that BGN4 potentially stimulates immune modulation via interaction of dendritic cells in the gut homeostasis.

As noted above, there are multiple experiments that provide evidence that BGN4 potentially affects the host immune systems and exerts protective actions from allergens through in vitro and in vivo studies, and show promising advances in the application of nutraceutical fields. Accumulating results indicate that some symptoms that are triggered by artificially treated allergens or antigens can be relieved by BGN4. Although physiologic outcomes have suggested possible benefits of BGN4, clinical efficacy of BGN4 has not been clearly established using single type of cell ingredient. Therefore, available evidence is not enough to demonstrate whether BGN4 may be more effective for bio-functionality in the human body than other microorganisms. Interpretation of the functional evidence of BGN4 is hampered by the presence of numerous other microorganisms. Further physiologic investigations are necessary to design formulations and to understand the basic mechanisms and bioavailability for studies of physiologic actions using single type of BGN4.

## 4. Anticancer Effects of *B. bifidum* BGN4

Colorectal cancer is a global health issue; in particular, South Korea showed the highest number of colon cancer cases in the world. As the third leading type of cancer in the world, approximately 1.4 million cases were identified in 2012 [85]. According to the American Cancer Society [86], colon cancer is the second leading cause of cancer mortalities with an expected 49,190 deaths in 2016. The international incidence and mortality rates of colorectal cancer are rapidly increasing in multiple countries as eating habits have been altered to a more occidental manner (i.e., low-dietary fibers, high-fat and high-protein) [87].

Various studies have reported that fermented food products can significantly prevent tumor growth by decreasing the risk of long-standing inflammatory responses in colon cancer. Probiotic microorganisms normally contained in fermented food products are known to offer functional effects on mucosal damages, specifically preventing the effects of cancer on the digestive tract [88]. Probiotics have multiple therapeutic advantages, playing significant roles in decreasing the mutagenicity of the epithelial layer, as reported in various experimental models of colorectal cancer [56]. In-depth investigations have shown that the relationship between colorectal cancer and probiotics seems to be primarily dependent on bioactive metabolites of probiotic bacteria, which lead to the generation of therapeutic anti-carcinogenic compounds [89].

Certain probiotic microorganisms and their extractions demonstrate growth inhibitory activities on adenocarcinoma cell lines. In particular, fractions from *Bifidobacterium* and *Lactobacillus* spp. containing high levels of microbial carbohydrates (i.e., extracellular glycoproteins, peptidoglycan, and polysaccharide) displayed profound tumor-suppressing activities [90,91,92]. These studies have demonstrated on evaluating the properties of probiotic fractions and extractions with regard to the decrease of viability or size of cell lines. However, proving the selective inhibition on cancer cells by probiotics treatment is critical with regard to the screening and selection of anti-carcinogenic substances. Moreover, anti-carcinogenic properties of probiotic microorganisms on colorectal cancer significantly differ from strain to strain, making it necessary to screen novel probiotic strains for tumor inhibitory effects [93]. Therefore, researchers should attempt to study the selectivity, sensitivity and specificity on noncancerous as well as cancerous cell lines using multiple probiotics.

Microbial polysaccharides are produced by probiotic bacteria with various health-promoting effectiveness. Their chemical structures, complexity and molecular weights differ among the probiotic species, resulting in expression of different physicochemical characteristics in both in vitro and in vivo systems. The antagonistic properties of probiotic bacteria against gastrointestinal illneses have been the subject of many clinical investigations, demonstrating varied functional properties. According to Nagaoka et al. [94], probiotic polysaccharides extracted from *B. breve* YIT4014 and 4043, and *B. bifidum* YIT4007 have shown anti-ulcer activities both directly (i.e., epidermal and fibroblast growth factor) and indirectly (e.g., immune system stimulating: increased production of 6-keto-prostaglandin F1 α by macrophage). Specifically, polysaccharides containing rhamnose as a major content (more than 60%) showed greater effectiveness in healing gastric ulcers. The soluble polysaccharides produced from *L. acidophilus* 606 also expressed the inhibitory effects on progression of colorectal cancer cell lines such as HeLa, PANC-1 and HT-29 cells and partially induced apoptosis into the HT-29 [90]. However, the polysaccharides from *L. acidophilus* 606 exhibited minimum toxicity into healthy human embryo fibroblasts.

These findings provided motivation to our group for observation and hypothesis on the correlation between BGN4 treatments and human colon cancer. Ku et al. [27] and You et al. [36] have demonstrated the tumor-suppressing activity of whole cell and its fractions of BGN4 on diverse adenocarcinoma cell lines. They also attempted to clarify whether such properties were human colon cancer cell-specific. Among the 30 kinds of different strains of *Bifidobacterium* tested in these studies, BGN4 showed the greatest anti-proliferative effects on human colon cancer cell lines. In particular, the polysaccharide fractions comprising chiroinositol, rhamnose, glucose, galactose, and ribose that were extracted from BGN4 induced significant growth inhibition of cancer cell lines (i.e., HT-29 and HCT-116), but did not show any growth inhibition of FHC (normal human colon cell) or Caco-2 cells, which are generally used as control group because of similar physicochemical properties with normal cells [95].

Previously, Shim et al. [35] discussed health benefits of fermented soy milk by BGN4, and confirmed that dietary BGN4 decreases the size of azoxymethane-induced aberrant crypt foci in rats. The combination of soymilk and BGN4 showed significant synergistic effects on reducing the number of aberrant crypt foci. The size and number aberrant crypt focus are commonly used biomarkers for colorectal cancer in rodents [96] and often regarded as the earliest histopathologic lesion linked to colon cancers [97].

The simple assessments of a decreased cell proliferation and aberrant crypt foci levels using in vitro and in vivo experiment are not enough to evaluate anti-cancer and/or anti-tumor properties of BGN4 due to the complexity of cancer development, which is linked to numerous cellular mechanisms. However, the anti-proliferation properties of BGN4 onto multiple cancer cell lines could be used as an example of the interaction between *Bifidobacterium* spp. and host. The interactions between BGN4 and colon cancer stimulated novel manners of cancer suppression and suggested the treatments of cell extractions with probiotic substances for the purpose of gaining anticancer properties. Further mechanistic studies and human epidemiological studies are necessary to elucidate the role of BGN4 and its extractable polysaccharides as a therapeutic option for anticancer or antitumor effects using animal models to take advantage of clinical properties derived from BGN4.

## 5. Industrial Application: Biocatalysis

Applications of health benefits from probiotics depend on the production of functional cell metabolites [5,7,52,56]. The commercial significance of health beneficial metabolites (i.e., polyssacchrides, bacteriocine, γ-Aminobutyric acid (GABA), and *S*-Adenosyl-l-Methionine (SAM)) from probiotic bacteria has stimulated the use of these bacteria as “Biological Factories” of value added products [98]. Bio-functional metabolites are produced by a variety of probiotic cells, especially *Lactobacillus* and *Bifidobacterium* spp., and have been intensively studied due to their broad spectrum of bio-functional properties and beneficial roles in human body. Due to the significance of probiotic cells in the expression properties of functional molecules, probiotic cells have been used in industry manufacturing for the production of value-added molecules [7,9,21,33,75].

Normally, the claimed functional benefits are likely achieved with high level of probiotic cells, however, multiple studies showed poor nutraceutical availability of some probiotic microorganisms in functional food products and found that they often exist at lower concentration in cells than those claimed on product packages [99]. Due to severe food processing and storage conditions (e.g., heat and acid treatment, artificial and natural preservatives, freeze and osmotic shock, and oxygen stress) often applied during the manufacturing step, maintaining cell activity and viability are practical challenge. Moreover, during the long-term period of circulation, viability of probiotic cells in product does not meet the criteria at the end of shelf life. Therefore, it is necessary to maintain cell viability and ensure probiotic effect for consumers’ needs [100]. A simple addition of probiotic cells into foods cannot guarantee health benefits to consumers. Therefore food industries have also encountered a number of difficulties when claiming the functional effects on the package of food products. For this reason, industry researchers and marketers have pursued to explore more applications of probiotics that can potentially be utilized in industry geared at several different markets [99]. Recently, various studies have proposed to use probiotic immobilization techniques to maintain microbial functionality and viability [101].

Extensive attention has been paid to the potential of using whole cell and/or cell fractions to facilitate the production of functional molecules [7,36,102]. Food and biotechnology industries have used advances in probiotics and their enzymes to produce value-added plant metabolites and/or their chemically transformed substances. Recently, bio-functional potentials of traditional herbal medicines and normal plants have emerged, resulting in notable progress in commercial developments of functional foods and/or nutraceuticals [41,102,103,104,105,106]. Specifically, to improve the quality of herbal resources, multiple probiotic cells and their enzymes have been applied for decades with commercial and domestic purpose in Korea under the concept of “fermented plant medicine” in the development and launch of nutraceuticals that is conceptually differentiated to other products [9,107].

This trend can be explained by favorable images of probiotics and herbal medicines among Korean consumers. According to Siró et al. [1], “Consumers need to understand the benefits, not the science behind the product”. Because of consumers’ limited understanding of functional foods and their health benefits, use of novel bio-functional materials could generate unnecessary work load and marketing cost to advertise and inform specific functional effects to consumers [99].

Phytochemicals are biologically and nutraceutically valuable plant metabolites. The isolation and recovery of target natural products from plants is often available for small quantities, specifically when small amounts of target molecules are naturally produced by plants and preserved in them [108]. Therefore, artificial pre-treatment (i.e., physical, chemical and enzymatic treatment) of phytochemicals to modify their chemical structures results in an increased yield of bioactive molecules and has conventionally been used to overcome limited supply issues. According to Gao et al. [107], biotransformation is “a chemical reaction that is catalyzed by whole cells (microorganisms, plant cells, animal cells), or by isolated enzymes due to high stereo- or regioselectivity combined with the high product purity and high enantiomeric excesses”. Bioavailable plant metabolites, specifically when they are in a glucoside form, are known to be functionally fortified by a deglycosylating process [9]. This biotransformation process selectively hydrolyzes target molecules and enables the structural conversion into valuable products. Biotransformation using biological catalysis can be carried out under relatively mild operational conditions compared to physical (heat treatment) reaction and/or chemical (i.e., acid and basic) catalyst counterparts, abridging the multifaceted manufacturing process [108,109,110]. Recently, biotransformation utilizing catalytic activity of microbial glycosidases has been recognized as useful technology in nutraceutical and pharmaceutical industries. Specifically, biotransformation of phytochemical glycosides using probiotic glycosyl hydrolases has played a great role in the production of bio-functional phytochemical aglycones with attractive potential for practical applications [102]. This bio-catalytic process using probiotic enzymes has been studied and applied as an essential manufacturing tool for enhancing the bio-functional and nutritional values of herbal medicines [107]. Through the fermentation process, plant glycosides can be catalyzed to aglycone, which has better bio-functional effects. Probiotic whole cells and their extracts (i.e., purified enzyme, cell-free extracts and crude homogenates) are increasingly utilized in the nutraceutical industry as key ingredients [9]. They have also been used as bio-catalytic agents that play a fundamental role in the bioconversion of herbal glycosides into aglycones induced by microbial enzymes belonging to different groups of glycosidases.

Panax ginseng, meaning “cure-all”, and its major functional metabolite, ginsenosides, are one of the widely-researched herbal medicines and phytochemicals [109]. There are multiple studies available covering various pharmaceutical properties of ginsenosides. In accordance with molecular mechanism, various pre-clinical and clinical studies have suggested that the regulatory properties of deglycosylated ginsenosides on diverse cellular mechanisms (i.e., cell cycle regulator (cyclin-dependent kinase), transcription factor (myc gene), signal protein (vascular permeability factor), tumor suppressors (cellular tumor antigen p53), cyclin-dependent kinase inhibitor (CDK-interacting protein 1), negative regulator of the p53 (mouse double minute 2 homolog) and apoptosis regulators (B-cell lymphoma-extra large, B-cell lymphoma-2 and X-linked inhibitor of apoptosis protein, etc.)) may have the anti-carcinogenic ability in prevention and management of cancer and chronic diseases [102]. Many studies have reported the phytoestrogenic properties of soy milk and soy isoflavones. Multiple evidences have shown that soy-derived phytoestrogens play inhibitory roles in osteoporosis, obesity and diabetes [111,112,113,114]. However, the functional effect of ginsenosides and soy isoflavones in in vivo systems are significantly dependent on enzymes of gut flora [115]. Therefore, merging probiotics and these phytochemicals may improve beneficial effects associated with intake of this plant medicine. Daidzein, genistein and glycitein that deglycosylated from of daidzin, genistin and glycitin have been introduced as chemo-preventive compounds for certain types of cancer (i.e., colon, breast and prostate) [116] and osteoporosis [117]. Similar to glycosylated ginsenosides, multiple studies demonstrated that the soy isoflavones in plants should be catalyzed into deglycosylated form (i.e., daidzein and genistein) for the effective absorption into blood stream across the gastrointestinal tissue [118]. The higher bioavailability after deglycosylation process has been demonstrated in in vivo experiments and explained by lower molecular weight and greater hydrophobicity than those of glycosidic compounds.

Because BGN4 naturally does not produce β-glucosidase during fermentation, soy isoflavones and ginsenoside glycosides cannot be catalyzed into the more bio-functional aglycones [24]. In this sense, BGN4 had practical limitation for industry application. Some researchers have manipulated expression vector to produce the recombinant BGN4 strain by cloning the structural β-glucosidase gene from naturally β-glucosidase producing *Bifidobacterium* spp. (i.e., *B. lactis* AD011, *B. lactis* SH5 and *B. lactis* RD68) [37,38,39,40,41]. β-glucosidase of *B. lactis* AD011 was cloned and overexpressed to apply ginsenoside conversion by Kim et al. [38]. BGN4 was employed as a sub-cloning and overexpression host for cloning of β-glucosidase of *B. lactis* AD011. However, BGN4 transformants (B141 and B893) could not use to catalyze artificial substrate (pNP-β-d-glucopyranoside) as well as natural substrates (ginsenosides). To attack this problem, Wang et al. [39] have attempted to highlight the necessity of powerful promoters for BGN4 in inducing significant expression of cloned genes with special focus on an exploration of the activity configurations of the promoters in BGN4. They proved that the activities of promoters were a function of microbial growth rates and that the use of P919 is effective to express of high-levels of foreign genes as a BGN4 promoter by hydrolyzing pNP-β-d-glucopyranoside into *p*-nitrophenyl and glucose. However, it should be considered that microbial β-glucosidases are quite unspecific catalysts whose specificities and activities possibly depend on a structural diversity of glycosides.

Recently, Youn et al. [37] highlights further how genetically-transformed BGN4 can be characterized as a β-glucosidase producer to practically apply for hydrolyzing natural products in which sugar moieties are linked to functional groups by a glycosidic bond (glycosides). They have constructed multiple expression vectors systems using bifidobacterial promoters (i.e., pamy, p919 and p572), ORF (i.e., bbg572), terminator (i.e., 572t) and signal sequences (i.e., ssamy) to produce new recombinant β-glucosidase-positive BGN4. The recombinant β-glucosidase, Bp572bbg572t was applied to catalyze multiple disaccharides (i.e., cellobiose, sophorose, laminaribiose and gentiobiose), Isoflavones (i.e., daidzin, genistin, and glycitin), ginsenosdies (i.e., Rb1 and Rb2) and Quercetins (i.e., isoquercetrin and spiraeoside) and successfully degraded glycosidic linkages between two molecules. This work would be practically useful for an application in phytochemical and bioconversion industries due to the understanding the vector systems/enzyme functions relationship. You et al. [41] also successfully carried out enzymatic catalysis of the isoflavone glycosides (i.e., daidzin, genistin and glycitin) into isoflavone aglycones (i.e., daidzein, genistein and glycitein) using a novel recombinant β-glucosidase. The β-Glu gene of *B. lactis* AD011 consisting of 1.4 kb was cloned and the recombinant β-glucosidase was overexpressed in BGN4.

As a result, BGN4 notably produced β-glucosidase and could be applied to convert ginsenoside glycosides and soy isoflavones into deglycosylated forms. The catalysis of plant glycosides using recombinant BGN4 would be applicable for commercial purposes [111,115]. The combination of this recombinant BGN4 with plant compounds could be utilized to produce fermented plant medicines with elevating amount of bioactive forms of ginsenosides and isoflavones. In addition, the synergistic effects generated by indigenous functional properties of BGN4 and its biogenic metabolites can be possibly expected. Recently, 110 Nobel Prize winners from diverse domains (i.e., medicine, economics, physics, chemistry, literature and peace) issued a statement in their support of modern genetic engineering, such as GMOs [119]. However, resistance to genetically-engineered probiotics from food consumers may exist in food market, resulting in the search for a new market (i.e., biomedical and pharmaceutical markets) exploitation beyond food or nutraceuticals, and consumers’ paradigm shift seems be necessary for successful commercial applications [1,9]. Before extensive utilization of genetically tailored BGN4 in nutraceutical products, consideration of possible safety issues and consumers’ prejudice is necessary.

## 6. Industrial Application: Bioactive Molecules

Among the various functional cell metabolites, GABA, a ubiquitous non-protein amino acid, is widely present in natural resources including bacteria, plants, and animals [8]. This molecule acts as a major inhibitory neurotransmitter in the brains of vertebrates and is produced by α-decarboxylation of glutamate by glutamate decarboxylase [120]. Recently, multiple studies have reported bio-functional effects of GABA (i.e., hypotensive, energy boosting, tranquilizing, lessens signs of aging, diuretic effects and anti-diabetes) [120,121]. Microbial glutamate decarboxylase, which is critical in GABA production, is widely distributed in probiotic cells. Multiple *Lactobacillus* spp. have expressed an ability to produce GABA in various levels depending upon the density of glutamates in the cell culture broth [122,123]. Recent reports have shown that *Gastrodia elata*, a traditional Asian plant medicine often applied for the treatment of neurodegenerative diseases and headaches, represses the degradation of GABA [124] and protects against neuronal damage. As a raw material, *Gastrodia elata* is regarded as a useful raw material for the GABA production due to the synergistic anti-hypertensive functions originated from *Gastrodia elata* [125].

To develop fermented *Gastrodia elata* products containing considerable amount of GABA, Kim et al. [30] applied *Lactobacillus brevis* GABA 100 and BGN4 simultaneously as a starter culture for *Gastrodia elata* fermentation. Previously, Kim et al. [126] reported high GABA-producing properties of *Lactobacillus brevis* GABA 100 that is isolated from Korean kimchi. The total GABA productivity was further increased by the co-culture of *L. brevis* GABA 100 with BGN4. The level of GABA observed during the co-culture was higher compared to the culture inoculated only by *L. brevis* GABA 100 by further decreasing media pH compared to its decrease in the single culture of *L. brevis* GABA 100. According to Komatsuzaki et al. [127], maintaining low pH (about 5) is necessary for effective GABA production. However, during the fermentation, normally the pH level of the cell culture media increased due to the enhanced level of GABA in the media.

As discussed above, due to the bioactive functionality of probiotic bacteria and its value-added metabolites, interest in mass production and industrial applications of biogenic molecules has been growing. Specifically, SAM, a commercially available and FDA-approved dietary supplement, has often been obtained and produced through chemical synthesis and cell fermentation. However, chemical synthesis has issues with production cost and generation of low purity products with optical isomers [128]. SAM, which is an amino acid naturally produced in the human body, plays a key role in transmethylation as a methyl donor. Multiple studies have extensively revealed the functional effects of SAM. As an important nutraceutical ingredient, SAM showed anti-depressant [129], anti-liver disease [130], and anti-headache effects [131]. According to Kim et al. [43], BGN4 produced a higher level of SAM compared to other microorganism. They used 25 kinds of different lactic acid bacteria (i.e., *Bifidobacterium*, *Enterococcus*, *Lactobacillus*, *Lactococcus*, *Pediococcus*, *Streptococcus* and *Weissella* spp.) and evaluated the productivity of SAM in culture media. The SAM productivity of BGN4 was at least two times higher than other bacterial strains. They also applied BGN4 to develop SAM-reinforced yogurt and reported favorable sensory value for commercial purposes [42]. However, little work was done to elucidate how productivity of above mentioned metabolites may be influenced by environmental factors including media ingredients, temperature and presence of other bacteria for commercial purposes. Nonetheless, above mentioned experimental data supports that BGN4 can be used in nutraceutical products as a microbial ingredient due to the benefits for human health.

## 7. Increase Biomass Productivity

For the commercialization, estimating productivities of cell and/or biogenic metabolites is necessary for cost-effectiveness of product manufacturing. In this sense, determination of appropriate media ingredients and formula design are important to enhance total cell-biomass productivity, as both biochemically and physiologically affect probiotic cultures [132]. When considering production costs of cell or biogenic molecules for commercial application, microbial enrichment for biomass production can be halted at the time of maximum productivity and some are left to run for longer, depending on the culture condition [133]. However, there are several pragmatic obstacles in mass production *Bifidobacterium* spp. and its metabolites for commercialization due to: (i) lower cell productivity after enrichment; and (ii) higher production cost compared to other aerobic or facultative anaerobic cells.

Kwon et al. [134] worked to develop a strategy to obtain high BGN4 biomass with greater metabolic products by combination of crossflow filter and cell reactor. Specifically, they submerged hollow fiber membrane (0.4 µm cut off, polyvinylidene fluoride, surface area of 25 m^2^) bioreactor with suction and gas sparging to maintain anaerobic conditions. By using this method, they were able to observe higher BGN4 viability and lower microbial harms generated by shear stress during crossflow filtration processes compared to conventional membrane reactor culturing. About 5 and 7 folds greater productivity of BGN4 cell biomass and viable cell counts (i.e., 12.0 g/L of biomass productivity and 2.2 × 10^10^ CFU/mL of maximum cell count) were observed when submerged hollow fiber membrane bioreactor was applied for BGN4 enrichment compared to the microorganism levels achieved through conventional batch culture (i.e., 4.5 g/L of biomass productivity and 3.0 × 10^9^ CFU/mL of maximum cell count).

Recently, Ku et al. [27] and Ji et al. [135] have reported more systematic approaches to increase BGN4 biomass productivity. They examined multiple factors, including: (i) media ingredients; (ii) types of acid; and (iii) incubation time when they utilized a two-step culture method that significantly affects the total recovery of BGN4 biomass and its bioactive metabolites. They reported that the phytic acid in culture media plays important role in improving the productivity and economic of BGN4 cell biomass and its biogenic molecules by changing microbial morphology and increasing size of the cell, although additional phytic acid treatments constitute a small portion from the overall costs of culture media formulation. These results agreed with various results showing that microbial shape and morphologies increase in the group in which media was treated with supplemented acids compared with those of control group [136,137,138,139]. It seems that the putative morphological modification effects of organic and mineral acids are likely due to their ability to act as cation chelators to induce response of the microbial mesosome control [140].

Even though multiple researchers have observed the putative role of acids as major inducer for the morphological modification of various bacteria, the molecular mechanisms underlying the producing properties of cell biomass are still largely unknown. Because chromosome sequencing data is available for BGN4, this information can be potentially utilized for designing better conditions of the biomass recovery than is currently possible. Moreover, transcription profiling over the fermentation procedures will provide information for stress and acid tolerance genes for BGN4. Additional work is necessary to better understand how genetic characteristics of BGN4 affect the recovery and production of BGN4 and its bioactive molecules using post-genomic approaches.

## 8. From Comparative Genomics to Functionality of BGN4

The *Bifidobacterium* genus is currently comprised of 47 recognized taxa, which have been isolated from six different ecological environments including the gut and oral cavity of human and animals insect hindgut, sewage and fermented foods [141,142,143]. The *Bifidobacterium* taxa can be clustered into six different phylogenetic taxa: *B. adolescentis*, *B. longum*, *B. pseudolongum*, *B. boum*, *B. pullorum*, and *B. asteroids*groups [144]. Although *B. bifidum* species have been represented one of the dominant bacteria from the gastrointestinal tract of breast milk-fed infants, *B. bifidum* is not included in six phylogenetic groups mentioned above, suggesting its unique and specific genomic composition [19,145].

The publicly available genome sequences to date contain three complete genomes of *B. bifidum* strains obtained from infant stool samples including BGN4 and 12 draft genome sequences (NCBI source). Among the genus *Bifidobacterium*, 23 complete bifidobacterial genome sequences are available. The size of *B. bifidum* genome is approximately 2.2 Mb (range, 2.14–2.28 Mb) and GC content is about 62% (Table 2).

In accordance with the analysis of other bifidobacterial taxa, enzymes in charge of the transport and metabolism of carbohydrates (Clusters of Orthologous Genes, COG category, G) were identified from the genome of BGN4 (Table 3), such as glycosyl hydrolases whose substrates are various oligo- and polysaccharides including human milk oligosaccharides (HMOs) and intestinal mucin [146,147]. Genome analysis of bifidobacterial species has revealed that the genus *B. bifidum* have adapted to ecological niches where there is a limited source of other nutrients except carbohydrates [148]. The *B. bifidum* pan-genome consisted of 2970 COGs and the core-genome was represented by 1295 genes. Those genes were dedicated to housekeeping functions of bacterial cells such as DNA replication, transcription and translation transport and metabolism of carbohydrates and amino acids, as well as host-interacting components of bacteria including sortase-dependent pili, tight adherence (*tad*) locus and murein lytic enzyme (*TgaA*). The pili structures reported to be crucial for interacting with host and other gut microbiota [141].

Duranti et al. [141] analyzed the average abundance of bifidobacterial DNA from 11 metagenomic datasets of the gut microbiome from infants and found that the relative abundance of *B. bifidum* DNA was 12.42% in breast milk-fed infants compared with the 0.24% in formula milk-fed infants. This result was consistent with the genomic analysis representing specialized catabolic ability of *B. bifidum* to utilize host glycans in an aspect of adapting to infant gut. Turroni et al. [146] also showed the specific ability of *B. bifidum* PRL2010 metabolizing host-derived glycans, especially HMOs and mucin. A comparative genomics study based on 15 genomes of *B. bifidum* strains helped to elucidate the evolutionary force for successful adaptation of this species to specific ecological niches (i.e., infant gut) by assessing genomic variability and complexity [141,142,147]. The genetic variability of *B. bifidum* was 13.7% of the total genomic pool of *B. bifidum*, and this was relatively lower than 15.3% of mobilome value in the genus *Bifidobacterium* [141,149].

In case of glycobiomes, *B. bifidum* showed a relatively small size of genes and especially reduced capabilities to catabolize high-molecular plant polysaccharides. However, it should be noted that *B. bifidum* contain enriched gene-sets undertaking the metabolism of host-derived glycans and health-beneficial glyco-conjugated phytochemicals. The comparative genomic analysis of BGN4 strain with other *B. bifidum* strains and whole bifidobacterial taxa has not been reported yet. Further study focusing on identification of unique BGN4 genes, which are capable of encoding specific colonizing factors, key enzymes catalyzing HMO and glycones, and immunomodulatory molecules expressed and secreted by BGN4, should be helpful to reinforce the multiple functionality of BGN4.

## 9. Conclusions

**Figure 2:**
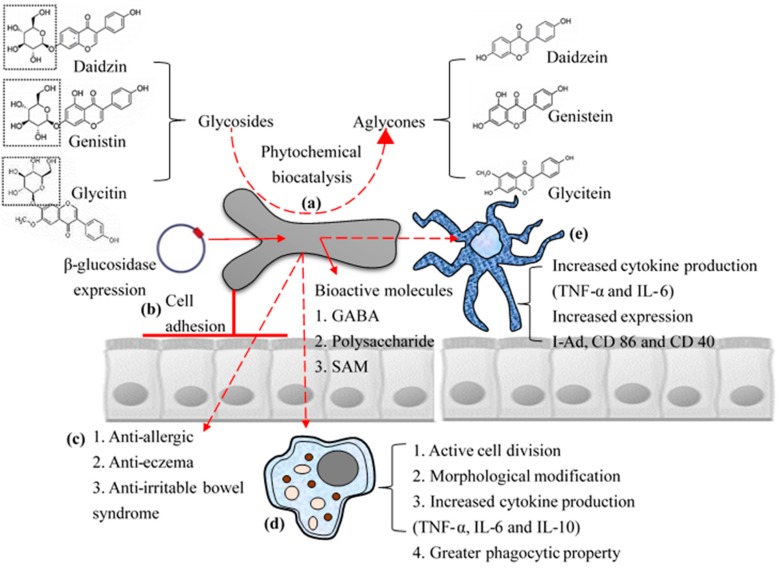
Schematic representation of biofunctional properties: Biotransformation of phytochemicals (**a**); high cell adhesion property with high surface hydrophobicity (**b**); and direct (**c**); and indirect immunomodulatory effects (activation of macrophages (**d**); and dendritic cells (**e**)) of *B. bifidum* BGN4 to host.

This systematic review summarizes bio-functionality of BGN4 assessed by in vitro, in vivo and clinical studies and potential of BGN4 for industrial applications and explains what is known and unknown based on available data (Figure 2). To demonstrate a precise mechanism of exploitation of BGN4 for human body, multifactorial clinical research and well-controlled molecular level work should be further pursued. However, potential functional value of BGN4 was clearly established through multiple in vitro and in vivo and clinical experiments. Moreover, BGN4 has been applied practically in commercial products with a mass production. Summarized information on BGN4 would be valuable to guide design with insight of future experiments to know mechanisms of functionality, clinical trials and commercial applications.

## Figures and Tables

**Table 1:** Microbial hydrophobicity of the cellular surface (CHS) among reference strains.

No.	Cell	CHS (%)	No.	Cell	CHS (%)
1	*B. bifidum* BGN4	93	20	*B. longum* ATCC 15707	<5
2	*Bifidobacterium* KJ	90	21	*B. longums* P-3	18.5
3	*Bifidobacterium* HJ-30	90	22	*B. animalis H-9*	37.13
4	*B. adolescentis* ATCC 15703	90	23	*B. animalis* P-4	17.4
5	*B. animalis* ATCC 2552	86	24	*B. asteroids* H-10	49.5
6	*B. animalis* M6	85	25	*B. pseudocatenulatum* I-6	47.3
7	*B. animalis* Rd60	69.6	26	*B. pseudolongum* CIDCA	85
8	*B. animalis* SI	66.3	27	*B. lactis* Bb12	75
9	*B. animalis* CN2	21	28	*L. acidophilus* LA5	75.1
10	*B. bifidum* ATCC 2952	12	29	*L. paracasei* (lac 1)	80
11	*B. bifidum* RD54	7	30	*L. acidophilus* (lac 2)	65
12	*B. bifidum* MS1	6	31	*L. acidophilus* (lac 3)	60
13	*B. bifidum* SH5	6	32	*L. acidophilus* (lac 4)	30
14	*B. bifidum* E15	5	33	*L. fermentum* (lac 5)	45
15	*B. bifidum* E2-18	<5	34	*L. fermentum* (lac 6)	65
16	*B. bifidum* JS9	<5	35	*L. acidophilus*	80
17	*B. bifidum* SH2	<5	36	*L. gasseri*	80
18	*B. bifidum* SJ32	<5	37	*L. jensenii*	80
19	*B. infantis* ATCC 15697	<5	-	-	-

The data number 1 to 20, 21 to 25, 26, 27 to 28, 29 to 34 and 35 to 37 were adapted from Kim et al. [26], Pan et al. [70], Pérez et al. [69], Shakirova et al. [68], Abdulla et al. [66] and Boris et al. [67], respectively. The level of CHS was evaluated by the cell adhesive method into xylene.

**Table 2:** Publicly available genome datasets of three different *B. bifidum* strains.

Strain Name	*B. bifidum* BGN4	*B. bifidum* PRL2010	*B. bifidum* S17
**Accession**	NC_017999.1	NC_014638.1	NC_014616.1
**Sequencing Status**	Complete	Complete	Complete
**Genome Size (bp)**	2,223,664	2,214,656	2,186,882
**G + C ratio (%)**	62.65	62.67	62.76
**Number of Chromosones**	1	1	1
**Number of Contigs**	1	1	1
**Number of ORFs**	1834	1706	1783
**Number of rRNA Genes**	9	9	9
**Number of tRNA Genes**	52	52	53

**Table 3:** Summary of genome analysis comparing COGs of three *B. bifidum* strains (analyzed by the authors based on NCBI datasets).

COG	Description	*B. bifidum* BGN4		*B. bifidum* PRL2010		*B. bifidum* S17	
		Number of Genes	%	Number of Genes	%	Number of Genes	%
***J***	Translation, ribosomal structure and biogenesis	136	10.56%	135	10.39%	135	10.48%
***K***	Transcription	95	7.38%	95	7.31%	93	7.22%
***L***	Replication, recombination and repair	102	7.92%	107	8.24%	100	7.76%
***D***	Cell cycle control, cell division, chromosome partitioning	24	1.86%	22	1.69%	23	1.79%
***O***	Posttranslational modification, protein turnover, chaperones	50	3.88%	50	3.85%	50	3.88%
***M***	Cell wall/membrane/envelope biogenesis	75	5.82%	81	6.24%	79	6.13%
***N***	Cell motility	6	0.47%	6	0.46%	5	0.39%
***P***	Inorganic ion transport and metabolism	50	3.88%	49	3.77%	49	3.80%
***T***	Signal transduction mechanisms	47	3.65%	50	3.85%	47	3.65%
***C***	Energy production and conversion	50	3.88%	50	3.85%	51	3.96%
***G***	Carbohydrate transport and metabolism	118	9.16%	117	9.01%	118	9.16%
***E***	Amino acid transport and metabolism	135	10.48%	137	10.55%	136	10.56%
***F***	Nucleotide transport and metabolism	56	4.35%	55	4.23%	56	4.35%
***H***	Coenzyme transport and metabolism	45	3.49%	44	3.39%	44	3.42%
***I***	Lipid transport and metabolism	35	2.72%	36	2.77%	36	2.80%
***Q***	Secondary metabolites biosynthesis, transport and catabolism	6	0.47%	7	0.54%	6	0.47%
***R***	General function prediction only	150	11.65%	148	11.39%	153	11.88%
***S***	Function unknown	108	8.39%	110	8.47%	107	8.31%
**Total**		1288	100%	1299	100%	1288	100%

## Abbreviations

BGN4	*Bifidobacterium bifidum* BGN4
CD	cluster of differentiation:
FDA	USA Food and Drug Administration
GABA	γ-Aminobutyric acid
IFN	Interferon
IL	Interleukin
SAM	*S*-Adenosyl-l-Methionine
SEM	scanning electron microscope
TNF	tumor necrosis factor
